# Supplementary material for: Attenuation of Pseudomonas aeruginosa Virulence by Pomegranate Peel Extract
Source: Microorganisms. 2022 Dec 16;10(12):2500. doi: 10.3390/microorganisms10122500 (PMC9784079; doi:10.3390/microorganisms10122500)
Supplement: Supplementary file 1 [file microorganisms-10-02500-s001.zip › microorganisms-2072922-supplementary.pdf]

Table S1: Peak areas of phenolic compounds in PomeGr exposed or not to *P. aeruginosa.*

Compound	*PomeGr peak area (AUP)*	*P. aeruginosa peak area (AUP)*	*(%) Decrease*
Galloyl-hexoside isomer 1	$7.82x10^6 \pm 2.71x10^5$	$7.57x10^6 \pm 4.24x10^5$	**3.2**
HHDP-hexoside isomer 1	$3.23x10^7 \pm 1.94x10^6$	$3.18x10^7 \pm 2.39x10^6$	**1.5**
Citric acid	$3.29x10^9 \pm 1.50x10^8$	$3.21x10^9 \pm 2.04x10^8$	**2.4**
HHDP-hexoside isomer 2	$8.47x10^7 \pm 1.98x10^6$	$6.46x10^7 \pm 1.76x10^6$	**23.7**
Galloyl-hexoside isomer 2	$1.35x10^7 \pm 2.03x10^6$	$1.54x10^7 \pm 5.52x10^5$	**14.1***
Galloyl-hexoside isomer 3	$1.02x10^7 \pm 3.40x10^5$	$1.00x10^7 \pm 5.94x10^5$	**2.0**
Pedunculagin isomer 1	$7.43x10^6 \pm 1.61x10^5$	$8.59x10^5 \pm 1.58x10^5$	**88.4**
Gallic acid	$5.55x10^7 \pm 2.61x10^6$	$5.20x10^7 \pm 5.83x10^6$	**6.3**
Galloyl-hexoside isomer 4	$1.29x10^7 \pm 3.59x10^5$	$1.26x10^7 \pm 1.10x10^5$	**2.3**
Galloyl-HHDP-hexoside isomer 1	$1.39x10^7 \pm 1.68x10^6$	$1.20x10^7 \pm 3.35x10^5$	**13.7**
Digalloyl-hexoside isomer 1	$1.09x10^7 \pm 7.88x10^5$	$1.05x10^7 \pm 2.99x10^5$	**3.7**
Punicalin	$6.48x10^7 \pm 2.40x10^6$	$5.11x10^7 \pm 5.00x10^6$	**21.1**
Pedunculagin isomer 2	$9.07x10^6 \pm 9.81x10^4$	$3.75x10^6 \pm 3.39x10^5$	**58.7**
Galloyl-HHDP-hexoside isomer 2	$1.25x10^7 \pm 1.42x10^6$	$8.88x10^6 \pm 4.25x10^5$	**29.0**
Punicalagin isomer 1	$3.70x10^7 \pm 1.23x10^6$	$2.44x10^7 \pm 4.16x10^5$	**34.1**
Digalloyl-hexoside isomer 2	$8.31x10^6 \pm 8.94x10^4$	$8.25x10^6 \pm 2.86x10^5$	**0.7**
Granatin isomer 1	$2.44x10^6 \pm 8.26x10^4$	$2.34x10^4 \pm 1.77x10^3$	**99.6**
Di-(HHDP-galloyl-hexoside)-pentoside	$5.56x10^6 \pm 2.70x10^5$	$9.76x10^5 \pm 4.56x10^4$	**82.4**
Granatin isomer 2	$1.47x10^6 \pm 7.41x10^4$	$7.59x10^4 \pm 3.10x10^3$	**94.8**
Punicalagin isomer 2	$3.61x10^7 \pm 1.02x10^6$	$1.01x10^7 \pm 4.07x10^5$	**72.0**
Punicalagin isomer 3	$8.04x10^7 \pm 8.61x10^5$	$5.46x10^7 \pm 7.67x10^5$	**32.1**
Brevifolin carboxylic acid	$9.55x10^7 \pm 2.09x10^6$	$9.47x10^7 \pm 3.44x10^6$	**0.8**
Galloyl-HHDP-hexoside isomer 3	$9.39x10^7 \pm 2.93x10^6$	$7.57x10^7 \pm 2.31x10^6$	**19.4**
Ellagic acid -hexoside	$1.93x10^7 \pm 3.28x10^5$	$1.95x10^7 \pm 5.88x10^5$	**n.d.**
Ellagic acid	$4.08x10^7 \pm 2.64x10^6$	$3.95x10^7 \pm 1.64x10^6$	**3.2**
Ellagic acid-deoxyhexoside	$5.21x10^6 \pm 1.51x10^5$	$5.02x10^6 \pm 2.34x10^5$	**3.6**

HHDP: hexahydroxydiphenoyl.

The relative amounts were expressed as the AUP (area under the peak) arbitrary units measured from the extracted ion chromatograms (EIC) obtained for each compound (tolerance ± 5 ppm).

n.d. non determined.

The asterisk indicates increase rather than decrease.

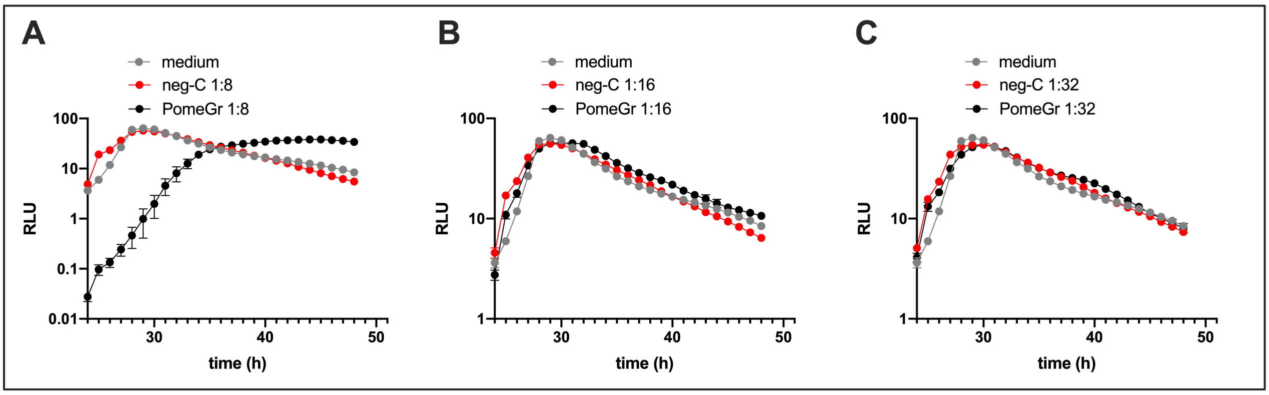

Figure S1: Kinetic of microbial regrowth. Twenty-four hours after exposure of Pseudomonas to PomeGr, neg-C or medium, the supernatants were removed and fresh medium was added to each well. The plate was further incubated at 37°C and the microbial regrowth was measured every hour (from 24 to 48 h). Panel A, 1:8 dilution; Panel B, 1:16 dilution; Panel C, 1:32 dilution. The results were expressed as mean ± SEM of the RLU of 9-12 replicates of three independent experiments.
